# Global Gender Disparities in Premature Death from Cardiovascular Disease, and Their Associations with Country Capacity for Noncommunicable Disease Prevention and Control

**DOI:** 10.3390/ijerph181910389

**Published:** 2021-10-02

**Authors:** Ji Zhang, Yinzi Jin, Peng Jia, Na Li, Zhi-Jie Zheng

**Affiliations:** 1 Department of Global Health, School of Public Health, Peking University, Beijing 100191, China; jizhang@bjmu.edu.cn (J.Z.); yzjin@bjmu.edu.cn (Y.J.); lina2021@bjmu.edu.cn (N.L.); 2 Institute for Global Health and Development, Peking University, Beijing 100871, China; 3 School of Resources and Environmental Science, Wuhan University, Wuhan 430072, China; jiapengff@hotmail.com; 4 International Institute of Spatial Lifecourse Epidemiology (ISLE), Wuhan University, Wuhan 430072, China

**Keywords:** cardiovascular disease, premature death, gender disparity, noncommunicable disease control

## Abstract

This study aimed to describe the prevalence of gender disparity in cardiovascular disease and explore its association with a country’s capacity for controlling noncommunicable diseases. Study data were extracted from the Global Health Estimates, and the Noncommunicable Disease Country Capacity Survey. Age-standardized premature death rates from cardiovascular disease, defined as any death occurring from ages 30 to 70 years, were calculated. Univariate and multivariate general linear regression models were fitted to estimate the correlations between gender disparity and country capacity for noncommunicable disease control. Globally, the premature death rate from cardiovascular diseases was 35.6% higher among men than women in 2000, and the figure hardly changed from 2000 to 2016. The highest gender differences were observed in Europe and high-income countries. The existence of dedicated and multisectoral noncommunicable disease governance bodies and the availability of cardiovascular disease stratification in primary healthcare facilities were positively correlated with gender differences. Conclusively, gender disparities in premature death rates from cardiovascular diseases differed with economic conditions and across geographic regions, with higher relative differences observed in more developed countries. The effects of existing control measures may have plateaued in men but are ongoing among women, especially in more developed countries, widening the gender disparity.

## 1. Introduction

Despite dramatic declines in mortality rates over the past decades in most countries and globally [1,2], cardiovascular disease (CVD) remains the leading cause of premature deaths for both genders [3]. CVD, including coronary heart disease and stroke, accounts for nearly half of all premature deaths globally [1,2], and the economic burden of the condition is considerable in both developed and developing settings [4,5,6]. In 2013, the member states of the World Health Organization (WHO) committed to achieving a 25% reduction in premature deaths from CVD and three other major noncommunicable diseases (NCDs) by 2025 [7]. Since then, national, regional and global progress has been reported each year. However, the gender disparities and temporal trends have rarely been discussed.

The incidence of, and mortality from, ischemic heart disease are higher in men than in women under the age of 65 years [1,8]. However, the gender differences narrow after 65, probably because women lose the protective effect of estrogen after menopause [1,8]. Furthermore, considerable evidence shows that CVD has been underdiagnosed, misdiagnosed, undertreated, and underreported in women [9,10] and that they do not receive proper preventive measures [8,11]. There may be a greater gender disparity in the probability of premature CVD deaths in higher-income regions [8], with different distributions of fatal and non-fatal disease burdens in different economic conditions. CVD death rates are decreasing for both genders but the rates of decline, including for CVD subtypes, may vary by gender [4,5,12,13]. Although the prevalence of CVD has been studied separately by gender, few studies have analyzed the gender difference as an indicator and discussed its related factors. A clear understanding of gender disparities in premature CVD mortality is essential in order to develop detailed and effective CVD prevention and control strategies.

CVD prevention and control have been implemented at individual, national, and global levels. Reducing existing modifiable risk factors, including tobacco use, the harmful use of alcohol, an unhealthy diet, and raised blood pressure, is a highly cost-effective way to reduce the NCD burden. In the conceptual framework of the social determinants of health, optimal policies at a national level can promote individual health behaviors so as to prevent and control the target disease. Therefore, integrated action at a national level, led by a well-functioning health system, is critical to achieve success. Since 2001, the WHO has therefore conducted a biennial assessment of the national capacity for NCD prevention and control, to monitor national progress, known as the NCD Country Capacity Survey (NCD-CCS). However, the global policy to boost and coordinate the actions in halving the incidence of CVD in women is reported to be inadequate [3]. 

This study therefore aimed to describe the premature death rate caused by CVD, any gender disparity, and the temporal trends regionally and globally. It also aimed to explore the association between national NCD control capacity and CVD gender disparity, to generate evidence that could help to develop more gender-specific recommendations for global strategies to achieve the global target for the reduction in CVD-related mortality.

## 2. Materials and Methods

### 2.1. Study Materials

#### 2.1.1. Global Health Estimates Country-Specific CVD Mortality

We used data on the age-, sex-, and country-specific deaths caused by CVD in the WHO Global Health Estimates from 2000, 2010, 2015, and 2016 [14]. The data provide a comprehensive and comparable assessment of mortality from diseases and injuries across all regions of the world, consistent with and incorporating UN agency, interagency and WHO estimates for population, births, all-cause deaths, and specific causes of death. The death causes were estimated from comprehensive death registration data, and detailed methods have been published online [15]. The data were downloaded in August 2020. No informed consent was required for this study.

For deaths from cardiovascular causes, the data extraction included three steps: extraction using a shortlist; the imputation of unknown cause–age and cause–sex groups with known ones; and the redistribution of ill-defined conditions. There were six subtypes of CVD: rheumatic heart disease (RHD), hypertensive heart disease (HHD), ischemic heart disease (IHD), stroke (including ischemic and hemorrhagic strokes), cardiomyopathy/myocarditis/endocarditis, and other circulatory diseases.

We defined premature death as death between the ages of 30 and 70 years [16] in this study. Age-standardized premature death rates caused by total CVD and each subtype were calculated using the standard populations [17]. The gender differences in premature deaths linked to CVD were derived by subtracting the death rate in women from that in men, and dividing by the death rate in men, to obtain the relative difference.

#### 2.1.2. Country Capacity for the Prevention and Control of NCDs

The WHO’s NCD-CCS questionnaire covers (1) health system infrastructurel; (2) funding; (3) policies, plans and strategies; (4) surveillance; (5) primary healthcare; and (6) partnerships and multilateral collaboration, although the precise content varies by year. The survey was carried out in 2001, 2005, 2010, 2013, 2015, 2017, and 2019, and details have been previously published [18]. We extracted six indicators related to CVD management, drawing on a framework of six building blocks of effective health systems [19]. These were as follows: (1) the existence of an operational unit, branch, or department in the ministry of health with the responsibility for NCDs; (2) the existence of a national multisectoral commission, agency, or mechanism for NCDs; (3) the existence of an operational, multisectoral national NCD policy, strategy, or action plan that integrates several NCDs and their risk factors; (4) the existence of an operational policy/strategy/action plan for cardiovascular diseases; (5) the availability of cardiovascular risk stratification in 50% or more primary healthcare facilities; and (6) the existence of a STEPS survey or comprehensive health examination survey every 5 years.

#### 2.1.3. Other Variables

Other national-level variables, including population size, gross domestic product (GDP), and health expenditure (% of total GDP), were downloaded in August 2020 from open data published by the World Bank (http://data.worldbank.org/, accessed on 20 September 2020). We used the WHO geographical classifications (Africa, America, South-East Asia, Europe, Eastern Mediterranean, and Western Pacific), and also categorized countries into four groups using the World Bank economic classifications: high-income countries (HICs), upper middle-income countries (UMICs), lower middle-income countries (LMICs), and low-income countries (LICs).

### 2.2. Statistical Analysis

We assessed age-standardized premature death rates linked to CVD and its subtypes, as well as gender differences for each country and for groups defined by the WHO geographical regions and the World Bank economic classifications. General linear regression models were fitted to estimate the correlations between national NCD control capacity and premature death rates in men and women, and their relative differences in 2015. Both univariate and multivariable regression models were fitted, with population size and income group as covariates in multivariate models. All statistical analyses used SAS 9.4 (SAS Institute Inc., Cary, NC, USA). All *p*-values were two-sided, and *p* < 0.05 was considered to be statistically significant.

## 3. Results

### 3.1. Regional and Temporal Trends in Total Age-Standardized Premature Death Rates

From 2000 to 2016, premature CVD-related death rates decreased from 113.2 to 82.9 overall, from 139.4 to 104.7 in men and from 88.9 to 63.0 in women (per 100,000 people), with significant temporal trends (annual percentage of change, APC = −1.9 in total, −1.8 in men, and −2.1 in women). Regional variations in premature death rates were also examined. CVD-related death rates in Africa, America, South-East Asia, Europe, Eastern Mediterranean, and Western Pacific regions were 121.75, 91.7, 115.42, 108.57, 136.71, and 114.08, respectively, per 100,000 people in 2000. The figures all decreased from 2000 to 2016, with insignificant temporal trends in South-East Asia and the Western Pacific region. America had the lowest premature death rates, and the Eastern Mediterranean had the highest. The mortality ranking was the same for men as overall. However, from 2000 to 2016, women in Europe had the lowest premature death rates from CVD, and African women had the highest (except in 2000, Table 1).

**Figure 1:**
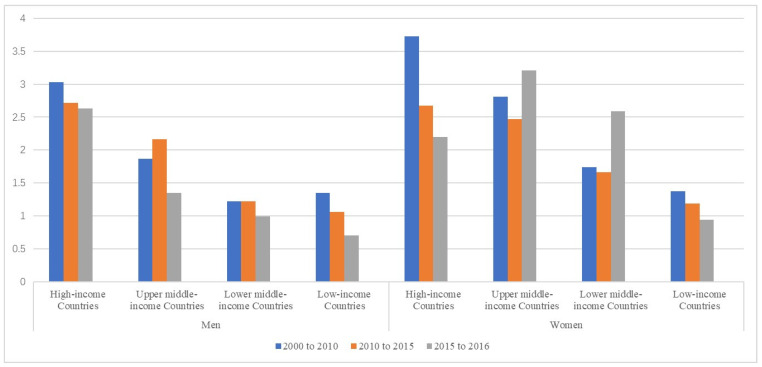
Percentage (%) of decline from 2000 to 2016 in premature CVD-related death rates, in men and women, by national income group.

From 2000 to 2016, the total premature CVD-related death rates were consistently lower in HICs than in UMICs, LMICs, and LICs, and the decreasing trends over time were all significant. The CVD mortality rates were higher in men than in women in all four income groups, although the decline in mortality varied (Table 2). In HICs and LICs, the mortality decline was similar in men and women. However, in UMICs and LMICs, the decline was greater among women than men (Figure 1).

IHD and stroke were consistently the leading causes of deaths from CVD in both men and women. There was little significant decline in premature deaths caused by HHD and cardiomyopathy in men. Premature deaths from RHD and HHD were higher in women than men in all data years, but the reverse was seen for other subtypes, including IHD, stroke, and cardiomyopathy. The temporal trends were statistically significant only for stroke, especially hemorrhagic stroke (Table S1).

### 3.2. Gender Disparities among Geographic Regions and Income Groups

Overall, premature deaths from CVD were 35.6% higher in men than women in 2000, and the figure barely changed from 2000 to 2016 (APC = 0.79, with an insignificant temporal trend). In 2000, the relative gender differences in Africa, America, South-East Asia, Europe, the Eastern Mediterranean, and the Western Pacific were 14.5%, 37.7%, 25.3%, 57.6%, 27.9%, and 39.9%, respectively (Table 1). The differences remained stable from 2000 to 2016 in Africa and the Eastern Mediterranean, but slightly increased in other regions, with the most significant change in South-East Asia (APC = 2.72). Over the six WHO regions and the four data years, the highest gender differences were seen in Europe, and the lowest in Africa (Table 1).

The highest gender differences were seen in HICs, with slightly decreasing trends from 2010 to 2016. In both UMICs and LMICs, the gender difference increased from 2000 to 2016, with APCs of 1.05 and 1.63. The gender difference in LICs remained the lowest, and hardly changed over the study period (Table 2).

**Figure 2:**
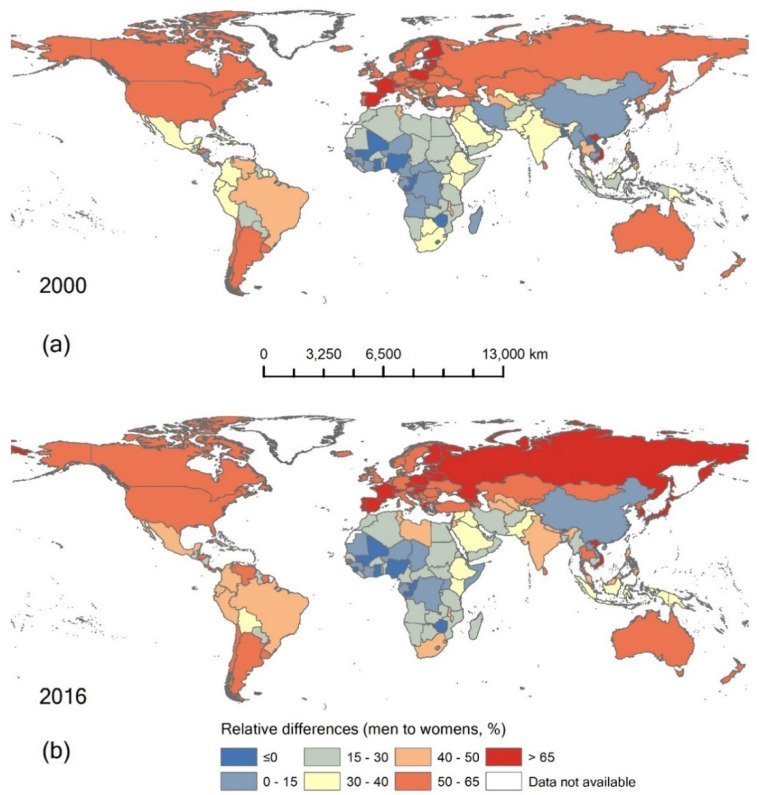
National variation in gender disparity in premature CVD-related death rates in (**a**) 2000 and (**b**) 2016.

Around 5% of WHO member states (eight countries in 2016, including Ghana, Mali, Sao Tome and Principe, Zimbabwe, Bhutan, Republic of the Congo, and Nigeria) had a higher premature death rate from CVDs in women than men. Countries with large gender differences were mainly in Europe, the Western Pacific region, and America. Most of the countries with small and negative gender difference were in Africa (Figure 2).

### 3.3. Premature Death Rates and Gender Differences Associated with National NCD Capacity of Prevention

In total, 167 member states (over 90%) responded to the NCD-CCS and had complete records for the six extracted indicators related to CVD. This included 51 HICs (30.5%), 47 UMICs (28.1%), 43 LMICs (25.7%), and 26 LICs (15.6%). Globally, 67.7% had an operational unit in the Ministry of Health with responsibility for NCDs; 34.1% had a national multisectoral commission for NCDs; 42.5% had an operational, multisectoral national NCD policy, strategy, or action plan that integrated several NCDs and their risk factors; 69.5% had an operational plan for CVDs; 22.2% had CVD risk stratification available in 50% or more primary healthcare facilities; and 21.6% had a STEPS survey or similar survey every 5 years (Table S2).

Most indicators were significantly associated with premature CVD mortality in women, but not in men, in univariate models. However, only the existence of an operational unit in the Ministry of Health with responsibility for NCDs was significantly associated with premature CVD mortality in multiple regression models (Table 3). Globally, four indicators were positively and statistically significantly associated with gender differences, including the existence of a dedicated operational unit in the Ministry of Health, a multisectoral mechanism for NCDs, the availability of CVD risk stratification in 50% or more primary healthcare facilities, and the existence of a comprehensive health examination survey. In the final multiple regression model, three of the six indicators (the existence of a dedicated operational unit in the Ministry of Health, a multisectoral mechanism for NCDs and the availability of CVD risk stratification in 50% or more primary healthcare facilities) were significantly associated with gender differences in premature CVD mortality, with coefficients of 15.03, 12.92, and 16.43 (Table 3).

## 4. Discussion

### 4.1. Overall Trends in Global Premature CVD-Related Deaths

Of the four major NCDs, CVD has been reported to be the most amenable to rapid change [7]. The younger population (i.e., those aged 30–70 years) is an important target for disease control because it is considered amenable to modifying behavioral risk factors, and therefore avoiding deaths [16,20,21]. We observed a significant decline in global premature CVD-related death rates (deaths in this population) from 2000 to 2016.

There were significant regional variations in premature CVD-related deaths and temporal trends. From 2000 to 2016, mortality rates decreased in all regions but to different extents. The largest decline was seen in Europe and the smallest was seen in South-East Asia. Consistently with other recent studies [22], the mortality declines were the largest in HICs. They also occurred in less developed countries, but to a lesser extent. The proportion of premature CVD-related deaths that occurred in HICs decreased from 16% in 2000 to 13% in 2016. However, the contribution from LMICs increased over that period. Premature mortality was higher in low-resource areas, such as Africa and LICs. Management and treatment were reported to be less advanced in these areas than in developed areas among both the pre-clinical population and diagnosed CVD patients [23,24]. It may therefore be helpful to focus more attention on less developed settings.

Of the nine voluntary targets [7] in the WHO NCD control action plan, two domains were risk factor control and national health system response. Previous studies have reported that a Mediterranean lifestyle was effective in NCD primary prevention, and this was recommended in the Eastern Mediterranean region [25,26]. However, overall mortality and men’s mortality were the highest in this region. Conversely, there were lower mortality rates in both genders and overall in Europe and America, indicating that effective measures to control CVD among both men and women may be established and implemented in those regions. The national capacity for NCD control may be an important factor in controlling NCDs in this region, and interventions targeting specific risk factors may not achieve the desired benefit if the healthcare system is not operating effectively. CVD prevention and control measurements may therefore operate in different ways at different socio-economic levels.

Across the six CVD subtypes, the largest declines were seen in IHD and stroke, which were also the leading contributors to CVD-related premature deaths. Over 80% of premature CVD-related deaths were attributed to these two conditions, and this has remained stable over the past two decades. Studies have demonstrated the disparities across genders [27] and regions [23] in IHD and stroke, and called for specific prevention and treatment guidelines and policies. We therefore examined these subtypes by gender.

### 4.2. Gender Differences in CVD-Related Premature Mortality

In recent decades, gender differences in CVD presentation, treatment, and outcomes have been recognized. Gender differences in the effects of controlling risk factors have also been found [20], indicating the need to identify the scale and causes of gender differences. This also affects priorities in controlling CVD. To our knowledge, this is the one of the first studies to map the global variation and temporal trends in gender differences in premature CVD mortality rates among WHO member states. Unlike the death rate, which has decreased, gender differences have remained stable over the past two decades, and mortality is one-third higher in men than in women (an average relative difference of around 35%). Gender differences also varied with the level of development, indicating an association with social, economic, or cultural factors, as well as biology. In each data year examined, the differences increased significantly with national income level and there was little change over time.

The construction of the variable means that a higher value can be interpreted as better cardiovascular health in women. In HICs and LICs, men and women shared similar rates of decline in premature CVD-related mortality, resulting in marginal changes in gender differences (<1%). In UMICs and LMICs, the decline in the mortality rate was higher in women than in men, and the gender differences showed more significant changes (>1%). In HICs, CVD mortality had declined for the past 50 years, and a slowdown had recently been reported in the rate of decline for both genders. This may be because these countries are reaching the limit of potential gains from reducing risk factors [28]. Studies have also reported more significant reductions in cardiac deaths in men than in women in the 2000s [29]. However, we found that the rate of decline may be slowing in men but appears to be ongoing for women, at least after 2010. Men are both the primary victims of CVD and the primary beneficiaries of control measures. CVD mortality among men started to substantially decrease 50 years ago, and the rate of decline has remained relatively high over a long period of time, although the pace has slowed more recently [28]. However, the death rate among women remained relatively steady during the implementation of early control measures but has fallen faster than that among men since 2000. The effect of efforts to reduce the burden of CVD on men may therefore have plateaued but is still ongoing in women, suggesting that the effect on women has lagged behind. In addition, health associations and initiatives began to focus on women’s cardiovascular health since the 2000s, first in the U.S. [30] and later in Europe [31]. These efforts to improve women’s cardiovascular health in highly developed countries caused the acceleration of the decline in mortality in those regions, and also demonstrated the necessity of gender-specific prevention measures. However, from the perspective of less developed countries or LICs, mainly in Africa and the Eastern Mediterranean, the gender differences have become smaller because of the relatively high mortalities in women and low mortalities in men. Due to the limitations of the study materials, we cannot assess the scale of biological differences between men and women, but we can recognize its existence according to previous studies. The reverse difference may suggest that healthcare focuses more on men in low-resource settings. We argue that gender differences in CVD mortality should be as close to the inherent biological differences as possible, rather than to numerical equality. Therefore, in less developed areas, policies and strategies for both genders should be fully considered.

In the conceptual framework of the social determinants of health, optimal policies at a national level can promote the individual health behaviors so as to prevent and control the disease. Although the prevention measures are eventually targeted at individuals, high-level policies are still critical to the process of delivery. For example, enhancing national investments in the screening and diagnosis of CVD risk factors among women can motivate women to take these tests and then promote the individual diagnosis and treatment of biological risk factors, or the correction of harmful behaviors. Furthermore, gender inequalities in healthcare are a consequence of basic inequality between men and women; therefore, multisectoral mechanisms could also play important roles in improving women’s cardiovascular health.

In addition to the shared contributors to mortality, women had a higher death rate from two of the six subtypes, RHD and HHD, which require more attention than those in men. RHD is a preventable disease, with greater effects in developing than developed countries. RHD is one of the major causes of maternal mortality, and is mostly preventable by reproductive health services in less developed areas [32]. There are proven prevention and treatment measures for RHD, but diagnosis and medication result in large medical expenses, which may only be addressed by means of an improved capacity and funding for a national health system. HHD is also one of the main contributors to women’s mortality. Women, especially those in less developed areas, appear to be bearing the brunt of RHD [33] and HHD [34].

### 4.3. National Capacity for the Prevention and Control of NCDs

Although women had lower CVD-related mortality than men, we know that CVDs in women are underdiagnosed and undertreated [3]. Women may therefore still suffer from poor management and healthcare. Women have also been reported to be more sensitive than men to some biological and behavioral risk factors associated with CVD, such as diabetes [35], obesity [36], and smoking [37]. These require decision-making and the implementation of actions by the healthcare system. The distribution of gender disparities across economic levels also supports this point. We therefore examined the effects of the national NCD control capacity on CVD gender differences.

The NCD-CCS contains no gender-related information. We therefore examined the whole-population national NCD capacity and its correlation with the death rates of both genders and the relative differences between them. We examined three domains: governance mechanisms, policies, and implementations, involving two of the six health system building blocks—governance and the delivery of health services [19].

Our study included two indicators reflecting the governance body: having a dedicated operational unit in the ministry of health and having a multisectoral mechanism. We found that the existence of an operational unit in the ministry of health with responsibility for NCDs is prevalent in many WHO member states, usually increasing with national income level. Only one-third of countries reported the existence of a national multisectoral mechanism for CVD control, including fewer than half of HICs and only one-fifth of LICs. Nearly 70% of countries had an operational strategy for CVD control, but fewer than half had an operational, multisectoral national strategy that integrated several NCDs and their risk factors. This suggests that CVD prevention and control may be a greater priority than that of other NCDs, at least at the national policy level. However, in health service delivery, CVD risk stratification in primary healthcare facilities and an NCD risk factor survey, such as the STEPS survey, were found to be much less prevalent. Only one-fifth of countries monitored the risk factors associated with CVD and other NCDs on a whole-population basis. Given the slowing decline in premature mortality in men, the positive correlation between gender differences and the national capacity indicators may be the result of the more significant effect of mortality reductions on women than on men. We have already seen that whole-population approaches seemed to benefit men earlier and more, and the benefits have only become apparent more recently in women. In recent decades, however, the effect has been greater among women.

We also found a positive correlation between gender differences and the existence in 2015 of both a dedicated NCD unit within the ministry of health and a multisectoral mechanism, with various levels of significance. Similar correlations were seen with CVD risk stratification in primary health facilities, but not with overall or CVD-specific policies. Dedicated and multisectoral governance bodies for NCDs are highly related to the security of the health workforce, health products, and financing as important factors in NCD control. CVD risk stratification also has more direct and significant effects on reducing premature CVD-related mortality than establishing policies. Therefore, the significant correlations may indicate the direct effects of the country’s capacity for prevention on improving women’s cardiovascular health, since the effects on men may have plateaued. Another study [23] showed that in many LMICs, the major barrier to reducing mortality may be obstacles to implementing guidelines and policies, rather than to establishing them. Experience from both HICs [38] and LICs [39] also shows that responses to NCDs must be comprehensive and multi-sectoral, involving actors at various levels, and covering both policy and implementation.

Modifying risk factors, therapeutic strategies, and overall policies beyond those strategies have been reported to be responsible for the reduction in cardiovascular deaths, with their relative contributions varying in different countries. However, the scale of decline seems smaller in less-developed countries. The comparison of the contributions of prevention measures to the mortality reductions across countries at different income levels would be an interesting topic for further study, and we are going to add some quantitative analysis into our ongoing researches. We also call for global responses to strengthen the capacity to both formulate and implement policies to address the widespread barriers to the management and treatment of CVD. The extent to which policy statements translate into implementation also needs to be evaluated.

## 5. Conclusions

We found geographic and temporal variations in gender differences in premature CVD-related mortality around the world. Our study suggests that there are higher relative differences (comparing men to women) in countries with a higher degree of development, suggesting that these countries have focused more on women’s cardiovascular health. The variation in gender differences also suggests the importance of social determinants in premature CVD mortality by gender. National NCD control capacity and its association with gender differences in premature CVD-related mortality rates suggest that the effects of current CVD prevention and control measures may have plateaued in men but are still working in women. We therefore call for the adherence to current policies and strengthening the implementation of these strategies, with additional efforts made towards cardiovascular health interventions for women.

## Figures and Tables

**Table 1:** Age-standardized premature CVD-related death rates and relative gender differences across geographic regions, from 2000 to 2016.

Region		2000	2010	2015	2016	N	APC	*p* for Trend
Relative Gender Differences (%)	Overall	35.6	39.0	39.8	40.5	183	0.79	0.177
		(32.2, 38.9)	(35.6, 42.4)	(36.4, 43.2)	(37.1, 43.9)			
	Eastern Mediterranean	27.9	30.0	30.3	30.5	21	0.07	0.855
		(22.2, 33.7)	(25.3, 34.6)	(25.9, 34.8)	(25.9, 35.2)			
	Europe	57.6	61.6	62.3	62.5	50	0.51	0.02
		(54.8, 60.4)	(59.0., 64.2)	(59.9, 64.6)	(60.2, 64.7)			
	Western Pacific	39.9	45.8	47.2	48.8	21	0.13	0.457
		(31.5, 48.3)	(37.0, 54.6)	(38.5, 55.9)	(40.0, 57.7)			
	South-East Asia	25.3	33.8	38.0	38.8	11	2.72	0.525
		(7.8, 42.9)	(18.1, 49.5)	(23.2, 52.9)	(23.7, 54)			
	Africa	14.5	15.7	15.5	16.5	47	0.66	0.971
		(8.5, 20.4)	(9.8, 21.6)	(9.1, 21.8)	(10.6, 22.5)			
	America	37.7	41.1	42.3	43.0	33	0.8	0.358
		(33.0, 42.4)	(36.3, 45.9)	(37.9, 46.6)	(38.5, 47.5)			
Men (per 100,000 people)	Overall	139.4	116.1	106.1	104.7	183	−1.79	<0.0001
		(130.3, 148.6)	(107.4, 124.7)	(98.4, 113.9)	(97.1, 112.3)			
	Eastern Mediterranean	158.2	131.1	121.3	120.5	21	−1.72	0.061
		(136.5, 179.9)	(107.5, 154.6)	(97.9, 144.6)	(97.1, 143.8)			
	Europe	155.2	124.8	107.1	103.6	50	−2.48	0.007
		(129.5, 180.9)	(99.7, 149.9)	(86.1, 128.1)	(83.4, 123.8)			
	Western Pacific	141.7	118.8	112.7	112.0	21	−1.48	0.376
		(110.4, 173)	(91.2, 146.5)	(85.6, 139.8)	(84.6, 139.4)			
	South-East Asia	135.0	125.4	120.2	118.9	11	−0.78	0.723
		(111.4, 158.6)	(101.7, 149.1)	(94.7, 145.7)	(93.8, 143.9)			
	Africa	132.7	113.3	105.5	105.0	47	−1.48	0.0001
		(122.2, 143.2)	(104.0, 122.6)	(96.3, 114.8)	(95.8, 114.2)			
	America	113.2	92.2	87.1	86.6	33	−1.69	0.016
		(98.1, 128.3)	(79.6, 104.8)	(73.8, 100.3)	(73.3, 99.9)			
Women (per 100,000 people)	Overall	88.9	70.9	64.4	63.0	183	−2.13	<0.0001
		(82.6, 95.3)	(65.1, 76.7)	(58.9, 70.0)	(57.5, 68.5)			
	Eastern Mediterranean	113.5	91.5	84.5	83.6	21	−1.91	0.072
		(94.8, 132.2)	(72.6, 110.5)	(65.9, 103.1)	(65.1, 102.1)			
	Europe	68.2	49.9	41.4	39.6	50	−3.32	0.0004
		(55.5, 80.8)	(38.9, 60.9)	(32.3, 50.4)	(31.0, 48.2)			
	Western Pacific	86.7	66.0	61.2	58.5	21	−2.37	0.099
		(64.6, 108.9)	(48.6, 83.3)	(44.8, 77.5)	(42.4, 74.7)			
	South-East Asia	98.1	82.4	74.4	73.0	11	−1.83	0.286
		(73.3, 122.9)	(60.5, 104.3)	(52.9, 95.9)	(51.4, 94.6)			
	Africa	111.2	94.6	88.2	87.0	47	−1.53	0.001
		(101.6, 120.9)	(85.0, 104.2)	(78.5, 97.8)	(77.3, 96.6)			
	America	71.4	55.2	51.6	50.6	33	−2.13	0.021
		(59.3, 83.5)	(45.2, 65.2)	(41.2, 62.1)	(40.2, 61.0)			
Total (per 100,000 people)	Overall	113.2	92.6	84.5	82.9	183	−1.93	<0.0001
		(106.1, 120.3)	(86.0, 99.1)	(78.4, 90.6)	(76.9, 88.9)			
	Eastern Mediterranean	136.7	112.4	104.0	103.1	21	−1.77	0.056
		(117.3, 156.1)	(91.7, 133.0)	(83.7, 124.4)	(82.9, 123.4)			
	Europe	108.6	84.5	71.9	69.3	50	−2.75	0.002
		(90.6, 126.6)	(67.7, 101.4)	(57.8, 86.0)	(55.8, 82.8)			
	Western Pacific	114.1	92.7	86.4	84.1	21	−1.86	0.188
		(88.1, 140.0)	(71.2, 114.2)	(65.6, 107.1)	(63.6, 104.6)			
	South-East Asia	115.4	103.5	96.4	94.9	11	−1.21	0.383
		(94.6, 136.3)	(84.1, 122.9)	(75.8, 117.1)	(74.4, 115.5)			
	Africa	121.8	103.3	96.4	95.5	47	−1.52	<0.0001
		(112.6, 130.9)	(94.4, 112.2)	(87.6, 105.2)	(86.7, 104.3)			
	America	91.7	73.1	69.0	67.9	33	−1.86	0.015
		(78.5, 104.9)	(62.2, 84.0)	(57.3, 80.6)	(56.4, 79.5)			

Abbreviations: APC, annual percentage of change.

**Table 2:** Age-standardized premature CVD-related death rates and relative gender differences across income groups, from 2000 to 2016.

Income Groups		2000	2010	2015	2016	N	APC	*p* for Trend
Relative Gender Differences (%)	HICs	54.5	58.1	58.0	57.8	52	0.38	0.543
		(50.4, 58.7)	(54.1, 62.2)	(54.0, 62.0)	(53.7, 61.8)			
	UMICs	39.3	44.2	45.7	46.8	54	1.05	0.06
		(35.5, 43.2)	(40.0, 48.4)	(41.4, 49.9)	(42.4, 51.1)			
	LMICs	20.4	24.0	25.4	27.0	46	1.63	0.618
		(13.4, 27.5)	(16.9, 31.1)	(17.8, 33.0)	(19.6, 34.3)			
	LICs	19.6	20.0	20.5	20.8	31	0.32	0.993
		(13.4, 25.9)	(14.5, 25.5)	(14.9, 26.0)	(15.2, 26.3)			
Men (per 100,000 people)	HICs	103.9	76.4	66.6	64.8	52	−2.91	<0.0001
		(89.4, 118.5)	(63.2, 89.6)	(55.3, 77.8)	(53.9, 75.7)			
	UMICs	156.8	129.9	116.4	114.9	54	−1.94	0.001
		(136.9, 176.7)	(112.3, 147.5)	(101.9, 131.0)	(100.8, 129.0)			
	LMICs	151.2	133.7	125.8	124.6	46	−1.21	0.063
		(134.6, 167.7)	(117.4, 150.1)	(110.7, 140.9)	(110.0, 139.2)			
	LICs	151.3	132.2	125.3	124.5	31	−1.23	0.047
		(134.7, 168)	(116.8, 147.5)	(110.4, 140.3)	(109.7, 139.2)			
Women (per 100,000 people)	HICs	47.2	32.3	28.2	27.6	52	−3.33	<0.0001
		(39.8, 54.5)	(26.2, 38.3)	(22.9, 33.4)	(22.4, 32.7)			
	UMICs	89.6	67.4	59.5	57.6	54	−2.71	<0.0001
		(81.0, 98.2)	(60.4, 74.4)	(53.0, 65.9)	(51.2, 63.9)			
	LMICs	113.9	95.6	87.9	85.6	46	−1.74	0.0002
		(103.4, 124.5)	(86.2, 105.0)	(78.5, 97.4)	(76.2, 95.1)			
	LICs	120.8	105.2	99.1	98.2	31	−1.3	0.077
		(105.5, 136.1)	(91.4, 119.1)	(85.8, 112.4)	(84.9, 111.5)			
Total (per 100,000 people)	HICs	74.2	53.8	47.3	45.9	52	−2.96	<0.0001
		(64.2, 84.2)	(44.8, 62.7)	(39.5, 55.1)	(38.4, 53.3)			
	UMICs	121.6	97.3	86.4	84.6	54	−2.25	<0.0001
		(108.7, 134.6)	(86.3, 108.3)	(77, 95.7)	(75.4, 93.7)			
	LMICs	132.4	113.7	106.0	104.1	46	−1.48	<0.0001
		(120.3, 144.4)	(102.2, 125.2)	(95.1, 116.9)	(93.6, 114.7)			
	LICs	135.4	118.1	111.6	110.7	31	−1.26	0.002
		(120.0, 150.8)	(104.1, 132.1)	(98.2, 125.0)	(97.3, 124.1)			

Abbreviations: HIC, high-income countries; UMICs, upper middle-income countries; LMIC, lower middle-income countries; LICs, low-income countries; APC, annual percentage of change.

**Table 3:** Associations between relative gender differences in CVD mortality and national NCD prevention capacity, 2015.

Indicators of National Capacity	Gender Differences				Mortality of Men				Mortality of Women			
	Unadjusted		Multivariable Model		Unadjusted		Multivariable Model		Unadjusted		Multivariable Model	
	β Coefficient	*p* Value	β Coefficient	*p* Value	β Coefficient	*p* Value	β Coefficient	*p* Value	β Coefficient	*p* Value	β Coefficient	*p* Value
Existence of an operational unit, Branch, or dept. in the ministry of health with responsibility for NCDs	15.03	<0.0001	10.08	0.0099	−19.25	0.0336	−13.8	0.1627	−28.37	<0.0001	−20.65	0.0015
Existence of a national multisectoral commission, agency or mechanism for NCDs	12.92	0.0006	7.98	0.0338	−2.66	0.7674	3.33	0.7259	−16.54	0.0079	−7.34	0.2341
Existence of an operational, multisectoral national NCD policy, strategy, or action plan that integrates several NCDs and their risk factors	4.00	0.2785	−1.31	0.7522	2.87	0.7392	11.36	0.284	−5.17	0.3914	6.63	0.3346
Existence of operational policy/strategy/action plan for cardiovascular diseases	7.14	0.0703	−1.54	0.7421	−9.76	0.2911	−7.93	0.5052	−15.93	0.0131	−5.76	0.4548
Availability of cardiovascular risk stratification in 50% or more primary health care facilities	16.43	0.0001	13.13	0.0021	−6.85	0.5049	−2.88	0.7882	−22.2	0.0017	−16.08	0.0216
Has a STEPS survey or a comprehensive health examination survey every 5 years	11.22	0.0107	4.96	0.245	−14.08	0.1735	−6.18	0.5687	−18.95	0.0083	−8.21	0.2433

## Data Availability

The data of this study are available in a public, open access repository (http://apps.who.int/gho/data/node.main.A858?lang=en, accessed on 20 September 2020).

## Supplementary Materials

The following are available online at https://www.mdpi.com/article/10.3390/ijerph181910389/s1, Table S1: Premature CVD-related death rates and relative gender differences by CVD subtype, from 2000 to 2016; Table S2: Distribution of national NCD capacity indicators by income group, 2015, Table S3: Differences of CVD mortality among indicators of national NCD prevention capacity, 2015.
