# Supplementary material for: Global Gender Disparities in Premature Death from Cardiovascular Disease, and Their Associations with Country Capacity for Noncommunicable Disease Prevention and Control
Source: Int J Environ Res Public Health. 2021 Oct 2;18(19):10389. doi: 10.3390/ijerph181910389 (PMC8507619; doi:10.3390/ijerph181910389)
Supplement: Supplementary file 1 [file ijerph-18-10389-s001.zip › ijerph-1375283-supplementary.pdf]

Table S1. Premature CVD-related death rates and relative gender differences by CVD subtype, from 2000 to 2016

		2000				2010				2015				2016					
		mean	95%CI		percentage of CVD deaths	mean	95%CI		percentage of CVD deaths	mean	95%CI		percentage of CVD deaths	mean	95%CI		percentage of CVD deaths	APC	*p* for trend
Relative Gender Differences (%)	Total CVD	35.6	32.2	38.9	NA	39.0	35.6	42.4	NA	39.8	36.4	43.2	NA	40.5	37.1	43.9	NA	0.8	0.177
	RHD	-42.8	-51.4	-34.3	NA	-37.1	-45.7	-28.6	NA	-33.1	-40.9	-25.3	NA	-31.5	-39.4	-23.7	NA	-1.8	0.220
	HHD	-32.6	-46.5	-18.7	NA	-21.6	-34.9	-8.3	NA	-21.5	-35.0	-8.0	NA	-18.0	-31.2	-4.8	NA	-3.2	0.464
	IHD	47.9	45.2	50.7	NA	49.9	46.9	52.9	NA	50.4	47.4	53.4	NA	51.1	48.1	54.0	NA	0.4	0.487
	Stroke	17.1	13.0	21.3	NA	22.6	18.5	26.8	NA	24.3	20.1	28.5	NA	24.8	20.5	29.0	NA	2.3	0.045
	Ischemic stroke	20.4	15.2	25.6	NA	26.5	21.6	31.3	NA	27.4	22.5	32.3	NA	27.9	23.0	32.9	NA	2.0	0.128
	Hemorrhagic stroke	14.9	10.8	18.9	NA	20.3	16.2	24.4	NA	22.4	18.3	26.5	NA	22.9	18.7	27.1	NA	2.8	0.025
	Cardiomyopathy	38.4	32.3	44.4	NA	43.3	38.9	47.6	NA	44.0	39.9	48.1	NA	44.0	39.5	48.4	NA	0.9	0.291
	Other CVDs	27.4	22.7	32.1	NA	29.0	24.5	33.5	NA	27.0	22.1	31.8	NA	27.2	22.5	31.9	NA	-0.1	0.929
Men (per 100,000 people)	Total CVD	139.4	130.3	148.6	NA	116.1	107.4	124.7	NA	106.1	98.4	113.9	NA	104.7	97.1	112.3	NA	-1.8	<.0001
	RHD	2.2	1.9	2.4	1.5	1.5	1.3	1.6	1.2	1.3	1.1	1.4	1.2	1.2	1.0	1.4	1.2	-3.6	<.0001
	HHD	5.1	4.4	5.9	3.7	4.6	3.9	5.2	3.9	4.4	3.7	5.0	4.1	4.3	3.7	5.0	4.1	-1.1	0.297
	IHD	80.3	74.0	86.6	57.6	66.9	61.1	72.7	57.6	60.9	55.7	66.0	57.4	60.1	55.0	65.1	57.4	-1.8	<.0001
	Stroke	38.7	35.6	41.7	27.7	31.6	28.8	34.5	27.3	28.7	26.0	31.4	27.0	28.3	25.6	30.9	27.0	-2.0	<.0001
	Ischemic stroke	12.8	11.5	14.1	9.2	10.4	9.3	11.5	8.9	9.2	8.3	10.2	8.7	9.1	8.2	10.0	8.7	-2.1	<.0001
	Hemorrhagic stroke	25.9	23.7	28.0	18.5	21.3	19.1	23.4	18.3	19.4	17.4	21.5	18.3	19.2	17.2	21.2	18.3	-1.9	<.0001
	Cardiomyopathy	4.5	3.8	5.3	3.3	4.3	3.5	5.1	3.7	4.2	3.5	4.8	3.9	4.1	3.5	4.8	3.9	-0.6	0.848
	Other CVDs	8.8	8.0	9.5	6.3	7.3	6.7	7.9	6.3	6.9	6.3	7.5	6.5	6.8	6.2	7.4	6.5	-1.6	<.0001
Women (per 100,000 people)	Total CVD	88.9	82.6	95.3	NA	70.9	65.1	76.7	NA	64.4	58.9	70.0	NA	63.0	57.5	68.5	NA	-2.1	<.0001
	RHD	2.7	2.4	3.1	3.1	1.8	1.6	2.1	2.6	1.5	1.3	1.8	2.4	1.5	1.3	1.7	2.4	-3.7	<.0001
	HHD	5.8	5.1	6.5	6.6	4.9	4.3	5.5	6.9	4.5	4.0	5.1	7.0	4.4	3.9	5.0	7.0	-1.7	0.006

	IHD	40.9	37.5	44.3	46.0	33.1	30.0	36.1	46.6	30.1	27.2	32.9	46.6	29.3	26.5	32.1	46.6	-2.1	<.0001
	Stroke	31.3	28.7	33.9	35.2	24.2	21.9	26.5	34.1	21.6	19.3	23.8	33.4	21.0	18.8	23.2	33.4	-2.5	<.0001
	Ischemic stroke	9.8	8.9	10.7	11.0	7.5	6.7	8.3	10.6	6.7	5.9	7.4	10.4	6.5	5.8	7.2	10.3	-2.5	<.0001
	Hemorrhagic stroke	21.5	19.6	23.5	24.2	16.7	15.0	18.4	23.5	14.9	13.3	16.5	23.1	14.5	12.9	16.1	23.1	-2.4	<.0001
	Cardiomyopathy	2.3	2.0	2.6	2.6	2.0	1.7	2.2	2.8	1.9	1.7	2.1	3.0	1.9	1.7	2.1	3.0	-1.2	0.099
	Other CVDs	6.1	5.5	6.7	6.8	5.1	4.5	5.6	7.1	5.0	4.4	5.5	7.7	4.9	4.4	5.4	7.8	-1.3	0.009
Total (per 100,000 people)	Total CVD	113.2	106.1	120.3	NA	92.6	86.0	99.1	NA	84.5	78.4	90.6	NA	82.9	76.9	88.9	NA	-1.9	<.0001
	RHD	2.4	2.1	2.7	2.1	1.6	1.4	1.8	1.7	1.4	1.2	1.6	1.6	1.3	1.1	1.5	1.6	-3.6	<.0001
	HHD	5.5	4.8	6.1	4.8	4.7	4.1	5.3	5.1	4.4	3.9	5.0	5.3	4.4	3.8	4.9	5.3	-1.4	0.042
	IHD	59.9	55.4	64.4	52.9	49.4	45.3	53.4	53.3	45.0	41.2	48.6	53.2	44.1	40.5	47.7	53.2	-1.9	<.0001
	Stroke	34.8	32.1	37.4	30.7	27.7	25.3	30.2	29.9	24.9	22.6	27.2	29.5	24.4	22.1	26.7	29.5	-2.2	<.0001
	Ischemic stroke	11.2	10.2	12.2	9.9	8.9	8.0	9.7	9.6	7.9	7.1	8.6	9.3	7.7	6.9	8.4	9.3	-2.3	<.0001
	Hemorrhagic stroke	23.6	21.7	25.6	20.8	18.9	17.0	20.7	20.4	17.0	15.3	18.8	20.1	16.7	15.0	18.4	20.2	-2.1	<.0001
	Cardiomyopathy	3.4	2.8	3.9	3.0	3.1	2.6	3.6	3.3	3.0	2.6	3.4	3.5	2.9	2.5	3.3	3.5	-0.8	0.577
	Other CVDs	7.3	6.7	8.0	6.5	6.1	5.6	6.7	6.6	5.9	5.4	6.4	7.0	5.8	5.3	6.3	7.0	-1.5	0.000

Abbreviations: RHD, rheumatic heart disease; HHD, hypertensive heart disease; IHD, ischemic heart disease; APC, annual percentage of change; NA, not applicable.

Table S2. Distribution of national NCD capacity indicators by income group, 2015

Income Group	HICs N=51		UMICs N=47		LMICs N=43		LICs N=26		Total N=167	
	N	%	N	%	N	%	N	%	N	%
Existence of an Operational Unit, Branch, or Dept. in Ministry of Health with responsibility for NCDs	43	84.3	37	78.7	22	51.2	11	42.3	113	67.7
Existence of a national multisectoral commission, agency or mechanism for NCDs	25	49	18	38.3	9	20.9	5	19.2	57	34.1
Existence of an operational, multisectoral national NCD policy, strategy or action plan that integrates several NCDs and their risk factors	26	51	21	44.7	14	32.6	10	38.5	71	42.5
Existence of operational policy/strategy/action plan for cardiovascular diseases	39	76.5	35	74.5	25	58.1	17	65.4	116	69.5
Availability of cardiovascular risk stratification in 50% or more primary health care facilities	22	43.1	11	23.4	2	4.7	2	7.7	37	22.2
Has a STEPS survey or a comprehensive health examination survey every 5 years	17	33.3	10	21.3	6	14	3	11.5	36	21.6

Abbreviations: HIC, high-income countries; UMICs, upper middle-income countries; LMIC, lower middle-income countries; LICs, low-income countries.

Table S3. Differences of CVD mortality among indicators of national NCD prevention capacity, 2015

Indicators of National Capacity		Men				Women			
		mean	95% CI		*p* value	mean	95% CI		*p* value
Existence of an Operational Unit, Branch, or Dept. in Ministry of Health with responsibility for NCDs	No	119.7	37.4	192.4	0.034	82.8	14.8	146.0	<0.0001
	Yes	100.5	31.2	227.5		54.5	11.3	118.0	
Existence of a national multisectoral commission, agency or mechanism for NCDs	No	107.6	35.3	209.4	0.767	69.3	11.3	144.3	0.008
	Yes	105.0	31.2	230.5		52.8	11.6	112.3	
Existence of an operational, multisectoral national NCD policy, strategy or action plan that integrates several NCDs and their risk factors	No	109.7	34.0	225.7	0.027	65.7	11.3	136.6	0.031
	Yes	76.9	31.2	190.7		43.3	11.6	96.1	
Existence of operational policy/strategy/action plan for cardiovascular diseases	No	113.5	30.3	192.4	0.291	74.7	10.6	146.0	0.013
	Yes	103.7	34.0	227.5		58.8	11.6	118.2	
Availability of cardiovascular risk stratification in 50% or more primary health care facilities	No	108.2	31.2	218.9	0.505	68.6	11.3	137.0	0.002
	Yes	101.4	34.4	230.5		46.4	13.4	106.2	
Has a STEPS survey or a comprehensive health examination survey every 5 years	No	109.8	31.5	225.7	0.174	67.7	11.6	137.0	0.008
	Yes	95.7	35.3	210.0		48.8	11.3	121.5	

T-test was conducted between groups of national capacity.
